# In Their Own Words: Treating Very Young *BRCA1/2* Mutation-Positive Women with Care and Caution

**DOI:** 10.1371/journal.pone.0087696

**Published:** 2014-02-28

**Authors:** Lindsey M. Hoskins, Allison Werner-Lin, Mark H. Greene

**Affiliations:** 1 Clinical Genetics Branch, National Cancer Institute, Bethesda, Maryland, United States of America; 2 Social Policy and Practice, University of Pennsylvania, Philadelphia, Pennsylvania, United States of America; Population Health and Preventive Medicine, Malaysia

## Abstract

**Purpose:**

Young women who have been identified as carrying a deleterious mutation in *BRCA1* or *BRCA2* face a unique set of challenges related to managing cancer risk during a demographically-dense stage of life. They may struggle with decision-making in the absence of clear age-specific guidelines for medical management and because they have not yet fully developed the capacity to make life-altering decisions confidently. This study sought a patient-centered perspective on the dilemmas faced by 18–24 year olds who completed *BRCA1/2* gene mutation testing prior to their 25^th^ birthdays.

**Patients and Method:**

This study integrated qualitative data from three independent investigations of *BRCA1/2*-positive women recruited through cancer risk clinics, hospital-based research centers, and online organizations. All 32 participants were women aged 21–25 who tested positive for a *BRCA1/2* gene mutation between 2 and 60 months prior to data collection. Investigators used techniques of grounded theory and interpretive description to conduct both within and cross-study analysis.

**Results:**

Participants expressed needs for (1) greater clarity in recommendations for screening and prevention before age 25, especially with consideration of early and regular exposure to radiation associated with mammography or to hormones used in birth control, and (2) ongoing contact with providers to discuss risk management protocols as they become available.

**Conclusions:**

Health care needs during the young adult years evolve with the cognitive capacity to address abrupt and pressing change. Specific needs of women in this population include a desire to balance autonomous decision-making with supportive guidance, a need for clear, accurate and consistent medical recommendations. Optimally, these women are best cared for by a team of genetically-oriented providers as part of a sustained program of ongoing support, rather than seen in an episodic, crisis-driven fashion. A discussion of insurance issues and provider-patient cultural differences is presented.

## Introduction

### Hereditary breast and ovarian cancer risk for women aged 18–24

Deleterious mutations in the *BRCA1* or *BRCA2* genes increase a woman's lifetime risk of breast and ovarian cancer. By age 70, approximately 60–70% and 45–55% of *BRCA1* and *BRCA2* mutation carriers will develop breast cancer, respectively; 40% and 20% of *BRCA1* and *BRCA2* mutation carriers will develop ovarian cancer [1]. By age 30, however, these risks are 3.4% and 1.5% for breast cancer, and 1–2% for ovarian cancer [2]–[4]. While these risks are high in *relative* terms (*i.e.*, compared with age-matched women from the general population), they may be viewed quite differently if presented in *absolute* terms (*e.g.*, by age 30, 95% will *not* develop breast or ovarian cancer).

*BRCA1/2* mutation testing may be offered once a woman reaches age 18 [5]. However, independent decision-making is often not well-established by this time in life [6]. Evidence-based approaches to management widely-used for older mutation carriers have not been developed (see Table 1), nor have clinical trials been undertaken for mutation-carriers <25 [7]. Women aged 18–24 who pursue *BRCA1/2* mutation testing may receive highly personal and emotionally-charged cancer risk information before they are able to confidently manage this risk. The typical primary care provider is often not equipped to bring a balanced, authoritative perspective to the extraordinary concerns faced by these women. Consequently, young carriers experience distress that is both quantitatively and qualitatively different from that of older mutation-positive women [8]–[9], as they adjust to their mutation status and consider limited proven risk management options.

**Table 1:** A Suggested Risk Management Strategy for *BRCA1/2* Mutation-Positive Women.

**BREAST CANCER RISK MANAGEMENT**
By age 30, breast cancer risk is 3.4% for *BRCA1* and 1.5% for *BRCA2* mutation-positive women
Breast self-examination [BSE] starting at either 18 or 20 years of age [27].
□ BSE not proven effective to detect early BC or to reduce mortality [28].
□ Low sensitivity to palpable breast abnormalities in young women may result in a false sense of security [28].
Clinical breast examinations [CBE] beginning between 20 and 25 [27].
□ Frequent biopsies may increase anxiety [16], [29]
Mammography and/or MRI between ages 25 and 30, or 5–10 years earlier than youngest age at first BC diagnosis in the family [27].
□ The relationship between ionizing radiation exposure and breast cancer risk in mutation carriers remains unclear [29]–[30].
□ High density of young women's breast tissue often makes mammograms diagnostically inconclusive [31].
□ Frequent biopsies may increase anxiety [32].
Tamoxifen
□ 50% reduction in the risk of breast cancer in high-risk women under age 50 [33]–[34].
□ Data from mutation carriers are sparse, but suggests similar benefits.
□ Medication-related toxicities (e.g., endometrial cancer, DVT, stroke, particularly in women above the age of 50) have discouraged more widespread use [35]
Risk reducing bilateral mastectomy (RRBM)
□ Lowers breast cancer risk by ∼95% for women without a breast cancer diagnosis [36]; the breast cancer risk post-RRBM is not zero.
□ Low acceptability for women who are single or dating [11], [16]
□ Long-term sequelae of RRBM unknown.
**OVARIAN CANCER RISK MANAGEMENT**
By age 30, ovarian cancer risk is 1–2% for *BRCA1/2* mutation-positive women
Ovarian Screening, starting either at age 30 or 5–10 years earlier than the earliest age of first diagnosis in the family [37].
1. Transvaginal ultrasound with color Doppler
2. CA-125 serum marker
3. Pelvic exam every six months
□ These methods are not proven to reduce morbidity or mortality from ovarian cancer.
Risk reducing salpingo-oophorectomy (RRSO)
□ Lowers ovarian cancer risk by 85% for women without a breast cancer diagnosis.
□ RRSO substantially lowers lifetime risk of breast cancer for premenopausal women [38], yet it increases lifetime risk of osteoporosis and heart disease [39].
□ Low tolerability in women who have generally not completed childbearing.
□ Recent recognition that a significant fraction of what has been called “ovarian cancer” originated, in fact, in the fallopian tubes underscores the importance of including the fallopian tubes when RRSO is performed [23], [40]
□ Hysterectomy is not routinely performed during RRSO because endometrial cancer is not considered part of the BRCA-related spectrum of cancers [41].
Tubal Ligation
□ 60% reduction in ovarian cancer risk in *BRCA1* carriers [42]
□ Finding not consistently reproduced from one study to the next
□ Preserves fertility options
____________________________________________________________________________
Oral Contraceptives
□ 50% reduction in sporadic ovarian cancer risk in the general population, with protective effect greater among long-term users
□ Similar reductions observed in *BRCA1/2* mutation carriers.
□ Concerns regarding possible increased risk of breast cancer, particularly for long-term users [22]

Most extant literature regarding psychosocial aspects of *BRCA1/2*-related risk aggregates participants across the lifespan in recruitment and data analysis, obscuring development of evidence-based risk management tailored to the unique developmental needs of the youngest consumers. We sought a patient-centered perspective on the dilemmas faced by 18- to 24-year-olds as they considered *BRCA1/2* genetic testing and risk management.

## Methods

### Study Participants

Data were drawn from three separate qualitative studies; each used a developmental frame to investigate the experiences of *BRCA1/2* mutation-carriers during their reproductive years. Beginning in 2003 and 2009, Werner-Lin [10]–[13] and Hoskins [14]–[17] independently recruited cancer-free, *BRCA1/2*-positive women aged 18–35 from the University of Chicago Cancer Risk Clinic and the National Cancer Institute, respectively, plus through national online organizations. All data for those studies was collected either via in-person interviews conducted in the Chicago metro area (Werner-Lin) or via telephone interview based in Bethesda, but targeting women from across the US (Hoskins) Investigators performed these investigations after approval by appropriate local Human Investigations Committees in accord with an assurance filed with and approved by the Department of Health and Human Services, where appropriate. Investigators obtained written informed consent from each participant.

Data from participants aged 18–27 who were ≤25 when they completed genetic testing were eligible for this secondary analysis. We chose to limit analysis to only women aged 25 or younger at the time of testing in order to understand the unique needs of these very young consumers of genetic testing services; due to the absence of clear age-specific guidelines for medical management and the complex and dense demographic shifts that occur during young adulthood (e.g., unpartnered vs. partnered, nulliparous vs. parous), we hypothesized that the needs of women in this age range would be qualitatively different from those of women in their later twenties and thirties. Alphanumeric identifiers were assigned to 26 eligible individuals (see Table 2) All underwent genetic testing between1997–2010. In 2011, a focus group was convened during the national meeting of a support/advocacy organization for women with increased breast and ovarian cancer risk [18]. English-speaking (due to constraints imposed by the researchers' language ability) women aged 18–27 who considered or completed *BRCA1/2* mutation testing prior to their 25^th^ birthday were eligible for participation. Six unaffected women aged 21–25 consented to participate.

**Table 2:** Participant Demographics (N = 32).

Age at Interview	23.2 (21–27)
*BRCA1* Positive	19
*BRCA2* Positive	13
Relationship Status	
Single	13
In a committed relationship	15
Engaged or married	4
Childbearing Status	
Had ≥1 child	2
Desired child(ren)	24
Did not want children or undecided	6
Pregnant	0
Completed or Scheduled Risk-Reducing Bilateral Mastectomy	5
Completed or Scheduled Risk-Reducing Salpingo-Oophorectomy	0
Breast or Ovarian Cancer Diagnoses	0

### Data Collection

#### In-depth interviews

Investigators independently collected in-depth, semi-structured interview data from 2004–2009. Investigators' interview guide elicited data-rich reports of: family experiences with cancer and genetic testing; impact on family, peer and romantic relationships; beliefs about how cancer risk influences individual development; family formation; and attitudes towards risk-reduction. Table 3 outlines codebook themes. Originally, these data were subsumed in each investigator's study cohort, and participants of all ages combined were analyzed as one group. Data from eligible subjects were re-analyzed to examine whether themes were similar to or different from those of the entire study cohort.

**Table 3:** Codebook Excerpt.

AXIAL CODES Theme = Navigation	DEFINITION & PARAMETERS OF CODES	SAMPLE PARTICIPANT QUOTES
**Expectations, Anticipating Change**	Captures participant conjecture, speculations, assumptions about their future lives, action and reactions in relation to cancer risk and family development.	*Automatically thought I was going to have to get rid of my breasts. I remember sitting in the shower with the shower running and sitting in there like I was taking a bath and holding my breasts and thinking, you're not worth it.*
**Exploring Options**	Captures the process through which participants learned about (either pro actively or passively) their option for genetic counseling or testing and cancer risk management.	*So I like to learn more and more about what I should be doing for prevention wise. Even though no one really could tell me because of my age. So that's frustrating.*
		*I'm still not sure what I want to do with my breasts.*
**Having a Plan**	Participants' expressions of behavioral intent, using prospective (future tense) language to describe alignment with specific courses of cancer risk management.	*I always wanted to be a young mom…and even more so now knowing that a lot of people with BRCA also take out their ovaries, but like I'm keeping these in until I have to take them out. I always thought that I would do that. But, now I would probably do it a lot sooner.*
**Making Decisions**	Participants' expressions of resolution, commitment to certain courses of action to manage cancer risk, including explanation of decision making process (or the lack there of) and rationalizations for those actions.	*And I don't want to be pushed to something that is not going to work out.*
		*I cried my eyes out a little bit that day when I found out the news and I decided to just take the step forward and go. They don't make me who I am and I'm just going to go ahead forth with this because at least I can do something about it now.*
**Taking Action**	Participant reports of steps complete towards their goals of minimizing cancer risk, engaging in protective health behaviors.	*I walked right into my ob-gyn's office and said I'm ready to get tested and they asked if – they said for insurance reasons, they asked me a couple of questions. And because I had Ashkenazi background and it got approved and that was it. I didn't have any counseling or anything before. I had that done afterwards. I did it little backwards.*
**Living with it**	Participant reports of the impact on identity, quality of life, relationships, and social networks of engaging with the ‘cancer world’ with respect to mutation status. Includes sequelae of *not* yet engaging in protective health behaviors. Includes watching loved ones cope with the impact of cancer risk, diagnosis, treatment, and mortality.	*I went through a couple of things, you know, some shock. Not shock, I don't know why. Maybe shock when you go – when you hear the statistics and the numbers, you know, it was not a good day when I found out.*
		*I'll be driving on the road – I drive a lot for work – and I will literally have daydreams that I had two kids. I'm gone. I have two kids and my husband is older and in therapy and I'm not there. That's pretty hard*

#### Focus group

Investigators jointly developed a focus group guide based on findings from their earlier studies to address perspectives on: learning about cancer risk before age 25; cancer risk-management; the impact of genetic testing on identity, sexuality, family and social life; and family formation. After consenting, each participant selected a pseudonym to identify herself and within eight weeks of the 90-minute focus group, all participants completed a family history phone interview.

### Data Analysis

All data were audio-recorded and professionally transcribed *verbatim*. Using the *constant comparative* method, investigators independently examined a subset of transcripts from each study to generate provisional codes. Once a working list of codes was established, all transcripts were reread and coded. Grounded theory techniques were used to facilitate agreement on a set of codes that best represented each dataset. Investigators approached the separate codebooks as three waves of data collection to capture changes to standard care protocols, public policy addressing insurance and discrimination concerns, and the social perceptions of genetic susceptibility during each time frame (*i.e.*, 2004, 2009, 2011)

## Findings

### Participant Demographics

Data from 32 women aged 21–27 (mean age = 23.2) were included in the current analysis. All were confirmed *BRCA1* (n = 19) or *BRCA2* (n = 13) mutation carriers. At the time of initial data collection, 13 women were single, 15 were in relationships, and 4 were engaged/married. Two had children, 24 planned to have children, and six did not want children or were undecided. None had developed breast or ovarian cancer. None were pregnant, and none reported pursuing genetic testing to inform family planning. The majority had made active lifestyle choices to support healthy living since learning their mutation status. Five had completed risk-reducing bilateral mastectomy (RRBM) or had the procedure scheduled in the coming months; none had scheduled or completed risk-reducing salpingo-oophorectomy (RRSO).

### Provider recommendations

Overall, participants expressed satisfaction with the care they received from the variety of providers with whom they had contact during the process of genetic testing and initiation of risk management. Participants valued providers who acknowledged their priorities, fears, and obligations. Numbers after each participant pseudonym indicate age at the time of data collection. **Reza** (25) shared:

*A doctor [said], “Right now there's really no reason to (have a mastectomy) In the next few years there's probably going to be advances. I'm not telling you to wait fifteen years…” Because in my head it was, why should I wait until after I get cancer? But he was, like, “Wait till you're thirty.” And I was, like, “Okay, that makes sense to me.”*

**Pam's** mother was diagnosed with breast cancer at age 32, so Pam (22) initiated screening at 21 [19]. She was satisfied with her providers' dialogue about risk-reducing surgery, reporting:

*“They said the breasts have to go by 25. And between 30 and 35 the ovaries would need to go. I went to the surgeon and talked to her about it, and I gave her my reasons for not wanting to wait, she agreed with me. Which was nice.”*

#### Needing guidance and autonomy

Nineteen participants knew of the *BRCA1/2* mutation in their family prior to their own genetic testing. Yet, many found it difficult to have their requests for screening adequately addressed. **Monique** (25) remembered, *“…everybody was like, ‘oh, you're too young to know what you want.’… the primary response was never, ‘no, you shouldn't do it because medically it's not necessary.”*

Incompletely developed genetic and health literacy led several participants to misunderstand their risk of inheriting a mutation, cancer risk estimates associated with being a mutation carrier, or the residual risk of cancer post surgical risk-reduction. **Linda** (23) completed genetic testing at her gynecologist's suggestion. She recalled receiving her test results:

*I got a phone call from my gynecologist saying, “I was afraid of this but you tested positive.” And I was like, “test positive for what? I have no idea what you're talking about.” And she was like, “for the genetic mutation.” Had I been smart I would have seen the genetics counselor first.*

A lack of clear expectations about genetic testing and the implications of one's carrier status complicated participants' interpretation of test results. **Hannah** (19) provided an example of a common misunderstanding of statistics at they relate to genetic risk. She reported:

*(T)he way the genetic counselor explained it to us … there's only like a 25% chance of us both having it. [Since I have it], it's less likely [my sister] has it.*

Hannah did not understand that one sister's mutation status was unrelated to the other's individual probability of being a mutation carrier [20], [21]. Each child of a mutation-positive parent has a 50% chance of inheriting the mutation.

Participants acknowledged that incomplete health literacy increased their need for expert guidance. **Melissa** (27) stated, *“some of the research is conflicting concerning different things, and it sort of perturbed me that it wasn't presented to me that way. It was presented to me as ‘never take birth control.’”* Melissa wanted her providers to directly address the uncertainty regarding birth control pills. Yet, participants also valued autonomy in making choices; they wanted to weigh differing perspectives to come to a decision that best fit their own needs, rather than having their providers make choices for them.

#### Inconsistent, incomplete recommendations

Participants expressed frustration at receiving inconsistent, inaccurate, ambiguous or incomplete recommendations from providers regarding their cancer risk management. During the initial phase of their mutation-positive experiences, participants reported receiving information from genetic counselors, oncologists, obstetrician/gynecologists, breast surgeons, and general practitioners, each with her/his own discipline-specific perspective and knowledge base. **Hannah** reported, *“I never took birth control because, in the beginning, they thought those hormones could actually be really negative for me. Now I hear, ‘We were wrong. You should have been taking birth control all these years.’”* In the early years of managing women with *BRCA1/2* mutations, one major concern related to the possibility that exogenous hormones might further increase the risk of breast cancer – the most frequent syndrome-related malignancy. Newer data suggest that there is little evidence of increased breast cancer risk among mutation carriers using oral contraceptive formulations prescribed since 1975, while a 50% reduction in the risk of *BRCA*-related ovarian cancer is now widely accepted as an important benefit related to OC exposure [22].

Since *BRCA*-related male breast cancer penetrance is so much lower than that for females, providers and consumers often mistakenly believe hereditary risk of breast/ovarian cancer is passed only through the maternal bloodline. **Ruth** recalled a physician telling her that since cancer was present on the paternal side of her family, she *“[didn't] have anything to worry about, or maybe we'll start giving you a mammogram a little bit earlier than normal.”* **Isabelle** (22), whose mother developed breast cancer at age 25, was frustrated by her inability to access screening; despite a letter signed by a doctor confirming her need for MRI, another provider refused to start screening before she turned 25. Given her family history, she perceived this to be too late.

**Alysha** (26), who sought out a great deal of information online, shared *“(I was) very frustrated with the conflicting information that doctors were telling me, particularly about screening – when and how often and the consequences of various tests.”* Participation in the annual meeting of a consumer advocacy group validated her desire to gather information to feel well-informed. After attending the conference, she reported *“I feel much better informed about what I should be doing and what those types of screenings actually do and possible side effects and accuracy.”*

Genetic testing at age 18 confirmed **Lynn** (23) carried a mutation, yet she received the message from her physician that *“you don't need a mammogram at this age.”* This led her to feel out of place in the healthcare system and frustrated at her inability to access screening. Feeling “*paralyzed*” by this roadblock, she completed bilateral mastectomy at age 22, believing it to be the only way to effectively manage her cancer risk [17].

#### Family formation

Like older mutation-positive women in the two original study cohorts [11], [13], participants were concerned about the challenges presented by HBOC risk and family formation. They discussed the need to plan for varied ways to sequence risk-management and family formation plans to attend confidently to both. Several recalled providers who suggested that they advance family formation goals quickly to support risk-management. **Tracy** (26) recalled,

*I was talking to my genetic counselor, saying “I'm finally ready to get my breasts removed, who should I contact?” She found me the best surgeon in the state, who's wonderful. And she said “If you're still not interested in having kids, they've discovered tubal ligation can reduce your* [ovarian cancer] *risk. This might be a good option for you.”*

**Ashley** (24) recalled that her physician told her *“…to start thinking about having kids before I'm 30 because if you breastfeed before you're 30, it reduces your risk of breast cancer”* and she reported thinking, *“oh jeez, I gotta get moving here.”* **Charlotte** (25) recalled that physicians she saw in the context of a research study told her to *“start having kids at any time, get that done with..,”* contributing to her feeling of urgency to complete childbearing so that RRSO could be pursued. Although **Ashley** experienced this recommendation as informative and supportive, **Charlotte** was distressed by her perceived need to act quickly.

**Reza's** mother was in active chemotherapy treatment following her second ovarian cancer recurrence in three years. Reza (25) wanted to undergo RRSO, but her providers advised against it. She shared, *“Nobody would give me a reason that seemed real besides ‘you're too young, you'll want to have kids someday.”* **Sara** (22) also met with resistance about her decision to undergo RRSO, despite her certainty that she did not wish to have biological children. She lamented, *“I've gone to the doctor and expressed my fear of cancer. There's never been the acknowledgement of, ‘okay I hear you, you don't wanna have kids; if you wanna do surgery now, that's great.’”*

## Discussion

Barriers to effective utilization of the healthcare system to reduce mutation-related cancer risk lead young women to feel uncertain about risk-management, concerned about reproductive decisions, and pressured to make quick choices. Participants valued providers who addressed their priorities and obligations. They desperately wanted evidence-based guidance to inform their decisions, yet they reported receiving incomplete and inconsistent recommendations from providers with limited expertise in hereditary cancer risk assessment and management. Participants experienced being treated as too young both to know what they wanted and to make major healthcare decisions independently. Incomplete health literacy led many participants to overestimate their cancer risk, focusing on lifetime rather than shorter-term, and relative rather than absolute risk, indicating the need for providers with quantitative expertise in hereditary cancer genetics to facilitate ongoing education. The combination of inflated short-term risk perceptions, limited proven age-specific risk management strategies, and well-intentioned but inaccurate provider recommendations all created pressure to rush childbearing, so that surgical risk-reduction could be implemented. Utilization of services from genetically-oriented providers may support patients' acquisition of developmentally and medically sound knowledge of cancer risk *and* risk management. The Affordable Care Act of 2010, which requires group health plans to provide coverage for dependent children of policy-holders until their 26^th^ birthday, may levy additional pressure to decide early about risk-reducing surgery [24].

### Discrepancies in responding to cancer risk

Inaccurate cancer risk perceptions may increase distress in young women, cause them to delay genetic testing (if risk is inaccurately perceived as low), or to initiate risk-reducing surgery precipitously (if short-term risk is inaccurately perceived as very high) Rapid uptake of risk-reducing surgery following genetic testing, as seen in our sample, is driven by the perception that cancer risk is imminent. The need to accurately convey short-to-intermediate cancer risk (over 5–10 years) represents a major challenge.

Our data suggest discrepancies may exist between patient and provider responses to inherited cancer risk, further indicating the need for providers with expertise in both cancer genetics and the developmental needs of adolescents transitioning to adulthood. Providers may have difficulty believing that a 20-year-old faces similar risks to those observed in older mutation carriers. Trust in provider recommendations seemed to erode as differences between provider and consumer expectations grew. Providers may suggest women in their 20s postpone risk-reducing surgery until after childbearing to reinforce that imminent cancer risk is quite low, or because they assume young patients prioritize having children. Yet, recommendations linking risk management and family formation may signal to patients that physicians perceive short-term risk of cancer to be very high. When young adults are not ready to commit to life-long partnerships or to become parents, these well-intentioned recommendations are interpreted as a failure to be taken seriously. Striking generational and gender socialization differences may exist between patients and providers, leaving young women struggling to feel understood by those helping them make decisions about their care. Optimally, providers can convey the caring expected from an expert, while maintaining the neutrality necessary to guide patients confidently to individually-tailored and thoughtful decisions.

### Implications for practice

Ideally, young women would learn all the facets of risk management early in their experience as mutation carriers, allowing them to manage risk confidently. However, our data suggest that rapid developmental changes common during the transition to adulthood may necessitate a prolonged period of care for young women, whose needs are distinct from older mutation carriers for whom standard practice guidelines and clinical trials were designed. In the context of limited evidence, young women need to work methodically with providers who are knowledgeable and non-judgmental, to avoid rushing into decisions inconsistent with their life goals. Provider recommendations may be carefully tailored to attend to developmental readiness while still providing an integrated approach to care. Such a model may enable ongoing connection with patients as shifting life circumstances precipitate interest in more aggressive risk-reduction strategies.

### Limitations & Strengths

Participants received care over a thirteen-year period during which the treatment of *BRCA-*related cancer risk evolved. We maintained separate codebooks for each study to ensure temporal differences in standards of care and public policy were accounted for during analysis. All three studies recruited women who completed genetic testing before age 25. However, none recruited individuals aged 18–20 *at the time of data collection*. None enrolled racial or ethnic minority women, subpopulations which are under-represented in high-risk clinics. Uptake of genetic testing remains particularly low for women of African descent [25], and is further complicated by their higher incidence of genetic variants that are uninformative regarding risk stratification [26].

## Conclusions

Young *BRCA1/2* mutation carriers comprise a unique subset of high-risk women who require particularly thoughtful individualized expert care to meet their complex needs. Insuring accurate understanding of absolute short-term cancer risk is essential to informed decision-making.
